# Elastography Using Multi-Stream GPU: An Application to Online Tracked Ultrasound Elastography, In-Vivo and the da Vinci Surgical System

**DOI:** 10.1371/journal.pone.0115881

**Published:** 2014-12-26

**Authors:** Nishikant P. Deshmukh, Hyun Jae Kang, Seth D. Billings, Russell H. Taylor, Gregory D. Hager, Emad M. Boctor

**Affiliations:** 1 Department of Computer Science, The Johns Hopkins University, Baltimore, Maryland, United States of America; 2 Department of Radiology, The Johns Hopkins Medical Institutions, Baltimore, Maryland, United States of America; Rensselaer Polytechnic Institute, United States of America

## Abstract

A system for real-time ultrasound (US) elastography will advance interventions for the diagnosis and treatment of cancer by advancing methods such as thermal monitoring of tissue ablation. A multi-stream graphics processing unit (GPU) based accelerated normalized cross-correlation (NCC) elastography, with a maximum frame rate of 78 frames per second, is presented in this paper. A study of NCC window size is undertaken to determine the effect on frame rate and the quality of output elastography images. This paper also presents a novel system for Online Tracked Ultrasound Elastography (O-TRuE), which extends prior work on an offline method. By tracking the US probe with an electromagnetic (EM) tracker, the system selects in-plane radio frequency (RF) data frames for generating high quality elastograms. A novel method for evaluating the quality of an elastography output stream is presented, suggesting that O-TRuE generates more stable elastograms than generated by untracked, free-hand palpation. Since EM tracking cannot be used in all systems, an integration of real-time elastography and the da Vinci Surgical System is presented and evaluated for elastography stream quality based on our metric. The da Vinci surgical robot is outfitted with a laparoscopic US probe, and palpation motions are autonomously generated by customized software. It is found that a stable output stream can be achieved, which is affected by both the frequency and amplitude of palpation. The GPU framework is validated using data from in-vivo pig liver ablation; the generated elastography images identify the ablated region, outlined more clearly than in the corresponding B-mode US images.

## Introduction

Quasi-static elastography involves comparing pre-compression and post-compression ultrasound (US) images to measure the displacement of speckles [1]. This measurement is used to determine elasticity of the tissue, which is useful in distinguishing hard and soft areas [1]. Visualization of the strain map calculated from this displacement can help identify tissue features, such as malignant tumors [1]. This technique is commonly known as elastography [1]. Elastography can be used as an early diagnosis tool for cancer, where early detection is critical in reducing the number of cancer related deaths [2]. Elastography has been evaluated in human trials for breast [3], [4], prostate [5], [6], liver fibrosis [7], [8], ovarian [9], skin [10], and thyroid cancers [11], [12]. Thermal ablation monitoring involves ablating the cancer tumor with RF ablator; an ultrasound guided needle is placed near the target region predetermined by a CT scan [13]. An ablated region increases the stiffness of the burned tissue, which is easier to visualize in elastography [13]. Elastography helps to accurately position the needle near the target region with the assistance of B-mode images and to monitor the size of the burn [13]. Ablation needs to be stopped for the acquisition of RF data; this duration needs to be very small to maintain the target ablation curve [13]. Collection of this data and calculating elastography in real-time are challenges.

Newer ultrasound imaging techniques like shear wave elastography [14] (focused ultrasound induced shear wave) and vibro-elastography (external vibration with a mechanical excitation) [15] can generate very high frame rates of up to 10 kHz and 300 kHz respectively [14], [15]. These techniques also use correlation to measure elasticity; hence a very high speed matching engine is needed. These techniques require special devices and US machines to record the RF data. Additionally, these systems are expensive and not widely available; hence a low-cost and high performing elastography implementation is necessary.

Elastography is computationally expensive. Given the high acquisition speed of modern US systems, there is need for a real-time implementation of elastography. This paper details a novel complete system of GPU-based elastography. The first known elastography implementation of a general-purpose graphics processing unit (GPGPU, commonly known as GPU) was published by [16]. This implementation was based on time domain analysis of RF data. An implementation based on Fourier domain analysis was published in [17], where a hybrid CPU-GPGPU model was proposed. In this implementation the GPU computes displacement estimation using CUFFT library, whereas median filtering and strain estimation is performed by a CPU. This implementation [17] does not have a real-time pipeline to accept RF data from an acquisition system; moreover, the CPU implementation would increase the CPU utilization in an attempt to have a threaded model of this pipeline. This work was further extended to calculate the time constant estimator for visco-elasticity and poro-elastography [18]. Due to the CPU-GPGPU nature of the work, a threading model is difficult to synchronize and requires the stream scheduling capacity of Compute Unified Device Architecture (CUDA) [19].

Field programmable gates array (FPGA) and Digital signal processor (DSP) based implementations of elastography and ultrasound systems have been reported by [20]–[22]. FPGA are on-board chips which tightly integrate with the underlying ultrasound hardware, thereby helping these systems to obtain direct, rapid access to the raw data from the ultrasound transducers. This hardware is expensive and difficult to program. A GPU-based implementation is a much less expensive and more flexible option. Several ultrasound devices by companies such as Ultrasonix, Siemens, Philips, GE, Toshiba, Supersonic, and Hitachi come equipped with built-in elastography modules [23]–[25]. These machines generally use a CPU implementation, which puts a strain on the system resources. However, many of the existing ultrasound systems deployed around the world come equipped with external PCI express cards. In these cases, connecting an external GPU card to a machine is fairly straightforward.

Real-time feedback for intra-operative tasks needs fast elastography in order to correct the deformation caused by the movement of the organ, varying compression and the hand tremor of the operator [26]. Typically these corrections need calculation of multiple pairs of elastography from a pair of RF data. When tracking information is acquired, good RF pairs can be presorted by exploiting the geometric position of the probe with respect to a reference tracker. An EM tracked ultrasound elastography method has been introduced by [26]. The disadvantages are that EM trackers cannot be used in ferromagnetic environments. The use of robot controlled motion inducers is another option. Real-time elastography on the da Vinci robotic system and on a snake robot have been integrated in [27] and [28], respectively. This system generates a pre-defined palpation motion to generate a high quality elastogram, but relies on the assumption that the underlying organ is attached to a rigid body. This motion can be compensated by a high speed real-time system to generate high-quality elastogram.

### Contributions

We present an end-to-end real-time system which improves the speed of GPGPU-based implementation of normalized cross-correlation (NCC) elastography using the stream capability of CUDA. This real-time system receives radio frequency (RF) data from an ultrasound machine and processes it on a GPGPU to compute an elastography image. We designed our system to harness the CUDA stream and multiple instruction multiple data (MIMD) capability of modern GPGPU architectures. Typical elastography calculations involve several computationally intensive components including displacement map generation, post processing filters, strain calculation, dynamic range adjustment, and scan conversion. Each of these components is mapped to a CUDA kernel within the GPGPU. CUDA kernels are the basic parallelizable blocks in the CUDA programming language, similar to a function. Using CUDA stream functionality these kernels are connected to form an input-output pipeline. A CUDA stream ensures data integrity by limiting inter-component data access to within the pipeline, thereby enabling multiple CUDA streams to run in parallel. We present the benefit of our work through speed comparison of elastography on multi-stream GPU architecture, single-stream GPU architecture, and non-stream GPU architecture. The new system has achieved an elastography image generation rate of up to 78 frames per second, nearly matching the RF data acquisition rate of ultrasound machines. We further investigate the impact of NCC window size on both speed and quality of elastography images using in-vivo pig liver data.

To showcase the adaptability of our architecture we demonstrate two applications: real-time elastography by free-hand palpation using external tracking information (Online tracked ultrasound elastography (O-TRuE)), and integration with the da Vinci Surgical System for elastography by robot-assisted palpation. The original TRuE [26] method was an offline system where the RF data and tracking data was collected offline, timestamp synchronization was performed in matlab, frame selection was done using TRuE and finally the elastography was calculated for the chosen pair of RF data. There was no real-time feedback to the surgeons, and this problem was solved using O-TRuE method. O-TRuE is an end-to-end system which involves RF data acquisition from an ultrasound machine and tracking data acquisition from an EM tracking device, synchronizing these acquired data based on timestamp, passing this data to a selection engine which performs in-plane RF data frames search using TRuE, implementation of a queue mechanism to streamline TRuE calculation and elastography computation. Furthermore we devise a technique for output elastography image stream analysis, which we use to investigate improvement in the output stream quality of O-TRuE relative to untracked free-hand palpation. We also apply this analysis to evaluate the quality of output elastography images for different palpation motions generated by the da Vinci system. Finally we demonstrate how multiple O-TRuE images combined by weighted averaging produce a higher quality elastography image, which we analyze using contrast-to-noise ratio (CNR) and signal-to-noise ratio (SNR) values.

## Background

Several supporting systems and methods are used in the development of this work. This section briefly introduces the reader to these concepts.

### General Purpose Graphic Processing Units (GPGPU)

A GPGPU is composed of many core streaming processors working in synchronization with each other. Early models of GPGPU's were single instruction, multiple data (SIMD) processors, for which a single CUDA kernel executes on all cores at a given time [2]. Implementations based on this architecture were commonly limited by poor utilization of the GPGPU. Newer versions of GPGPU's, such as the Fermi-architecture from NVidia, resolve this problem by introducing multiple instructions, multiple data (MIMD) processors [19]. This paper exploits this new capability of the GPGPU by scheduling all elastography processing components into individual CUDA streams, which enables greater utilization of the GPGPU (henceforth referred to as GPU).

### Normalized Cross-Correlation (NCC) based Elastography

Normalized cross-correlation (NCC) is used for calculating tissue displacements between pre- and post- compressed RF data images by measuring the speckle shift [16], [29].
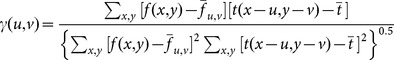
(1)


Equation 1 defines the NCC function for comparing two RF image regions where 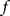 is a template window in the first RF image and *t* is a target window in the second RF image, 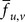 and 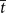 are the mean respective intensities within each window, and *x*, *y*, *u*, and *v* denote pixel position within a windowed region [29]. This window based approach allows processing individual window searched on separate CUDA threads [16]. A correlation map generated from eq. 1 is used to build a displacement map using cosine fit interpolation [16]. Median and moving average filters are applied on this displacement map to remove outliers [16]. The median filter and the moving average filter again allow for individual output pixels to be computed on different threads [16]. A strain map is finally generated from the displacement map using the least squares approach which can similarly be scheduled on individual threads [16].

### Tracked Ultrasound Elastography (TRuE)

Foroughi et al. [26] developed and validated TRuE on offline data using electromagnetic (EM) tracking of an ultrasound probe. As per [26], a cost function (eq. 2) is used to rank the quality of physical alignment between different RF data frame pairs, which is computed from the corresponding EM tracking data
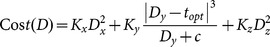
(2)where 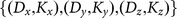 are the displacements and sensitivities of the motion in the lateral, axial and out-of-plane directions respectively, calculated using tracking information in the pair of RF data. A user input 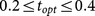
regulates the maximum displacement expected in the axial direction; 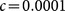 is a small constant to compensate for zero compression. The input D to the cost function is the distance vector 
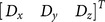
, which is calculated as follows:



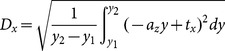
(3)

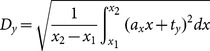
(4)

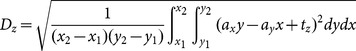
(5)where 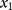, 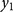, 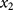 and 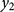 form a region of interest for each frame. The axis-angle representation of the rotation between frames is 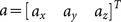
and the relative translation is 
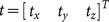
. In our paper we have used 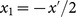,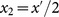, 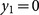 and 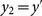, where 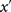 and 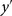 denote the image pixel width and height, respectively. These values are multiplied by pixel spacing values to convert to millimeter scale. Since we are primarily interested in axial motion analysis, the axial sensitivity is controlled as follows



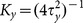
(6)

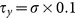
(7)where  is a user defined variable of type natural number in the range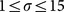 [26]. The value of 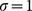 indicates lower sensitivity in the axial direction and  indicates higher sensitivity. Similarly, 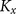 and 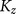 are defined as



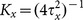
(8)

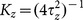
(9)where 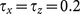 is fixed in our experiments since we are interested only in axial direction and the value of 0.2 can accommodate our small motions in lateral and out-of-plane directions. Finally, we define pseudo correlation value as the exponential of the negative cost value (eq. 2) as
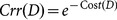
(10)which provides a value in the range [0, 1] to rank the quality of physical alignment between different RF frame pairs. RF frame pairs with high Crr values have image planes closely aligned in physical space, making them ideal candidates for elastography image computation. As previously described in discussion of eq. 2, from which Crr is derived, the Crr value is computed for an RF frame pair by analyzing the tracking information associated with each frame. This analysis is performed for all 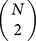 frame pair combinations amongst the N most recent RF frames stored in a buffer. The reader is referred to [26] for further details.

## Methods

This section describes our approach to GPU-based elastography. The system setup and configuration is presented in subsection *System Overview*. Subsection *Multi-Stream GPU-based Elastography* describes the architecture of the multi-stream implementation of GPU-based elastography. The online tracking implementation is discussed in subsection *Online Tracked Ultrasound Elastography*.

### Ethical Statement

Experiments were performed on a healthy pig as per the protocol number SW11M128 approved by Johns Hopkins University Institutional Care and Animal Use Committee. The experiments were conducted on pig liver since the pig liver is anatomically close to human liver. The experiment in-vivo is needed to reflect the conditions during surgery and validate the algorithm. The data for this paper was reused from earlier study in [26] to minimize animal experiments needed. We also extensively performed experiments on phantom to measure speed and define metric for O-TRuE to minimize experiment on animals.

### System Overview

**Figure 1:**
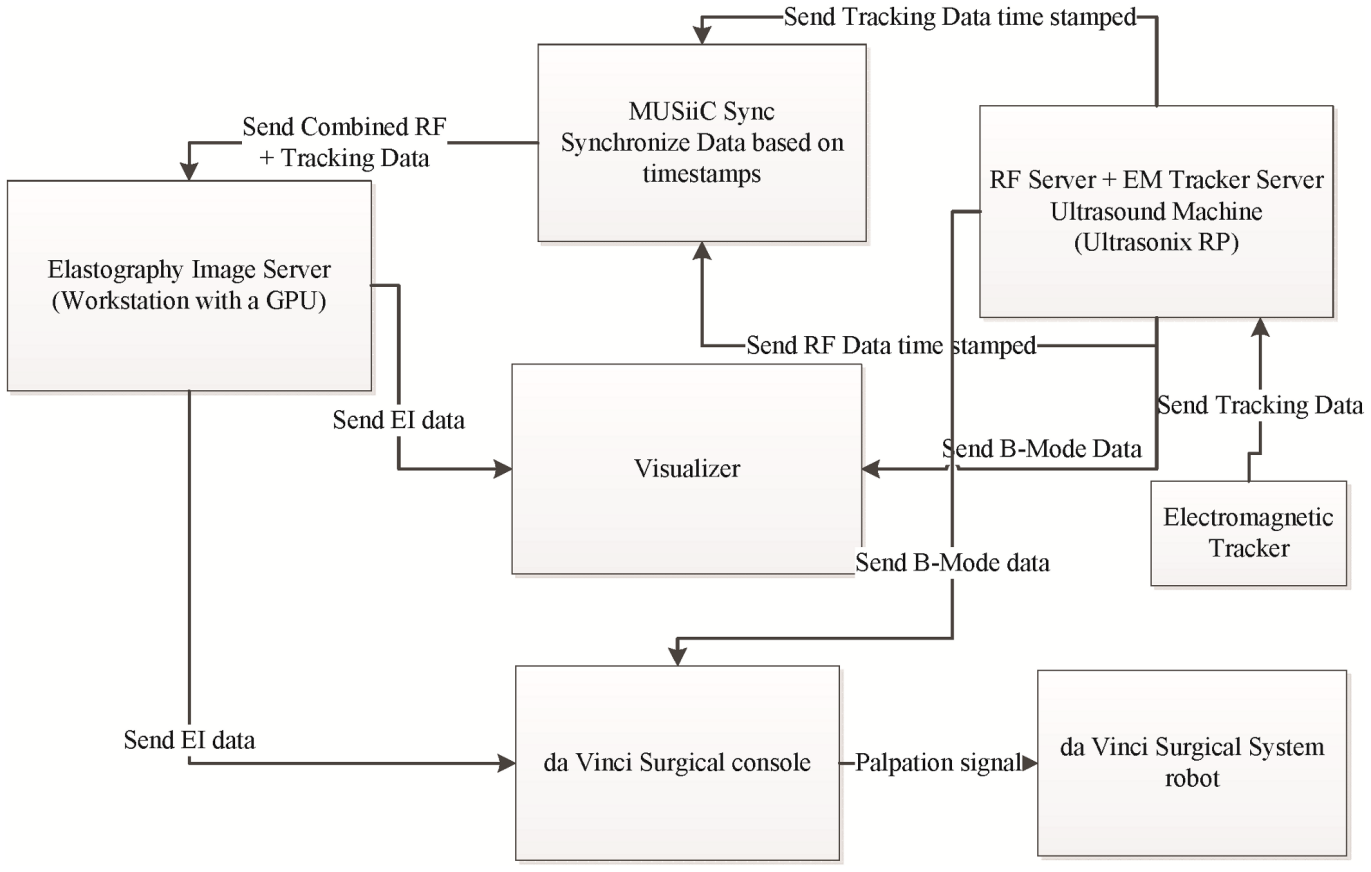
Overall System Diagram. The figure shows overall system and data flow diagram of elastography image server which runs on a machine equipped with a GPU. The system is modular with each module configurable to run on different machines or on a same machine (exception is hardware dependent da Vinci surgical system, RF Server and EM Tracker Server). The elastography image server is based on multi-stream elastography algorithm and with little change can handle both tracked and untracked RF data. The MUSiiC Sync synchronizes tracking and RF data based on timestamp to be processed by elastography image server. The system is flexible to be connected with da Vinci Surgical console to allow overlay of elastography and b-mode image stream.

Fig. 1 illustrates the overall system showing the various components comprising the real-time multi-stream GPU-based elastography system. This is an application view of the system by inclusion of the GPU, ultrasound machine, tracking system, da Vinci Surgical System, image visualizer, and the MUSiiC Sync application. Communication between all system components is accomplished using the OpenIGTLinkMUSiiC library [30]. This library assists to make the system highly modular, allowing deployment of the components on different machines. The MUSiiC Sync application serves to synchronize all time-stamped data sources within the system. In our configuration, the MUSiiC RF server and MUSiiC EM tracker server were run on the ultrasound machine in order to get synchronized timestamps. The MUSiiC RF server, which is part of MUSiiCToolkit [30], collects RF data from the ultrasound machine and sends it to the network. This data can be collected by several listening clients. Similarly, the MUSiiC EM tracker server sends real-time tracking information to the network. MUSiiC Sync synchronizes the RF and tracking data based on their timestamps and forms a single data packet from each synchronized data pair which is sent to the Elastography Image Server. The Elastography Image Server processes the synchronized data to choose RF data pairs for elastography computation, which it computes using GPU. Output elastography images are then sent from the Elastography Image Server to a visualizer.

The EM tracker, which is attached to the ultrasound probe, provides the position of the probe in 3D space. Applying TRuE to the input stream of position data gives the best frame selection capability. In the da Vinci Surgical System environment, where usage of an EM tracker is not possible due to presence of ferromagnetic materials, we rely on steady palpations generated by robot to grant a good quasi-static elastography [27]. When no tracking data is available, we do not need MUSiiC sync. In this case, the RF Server directly outputs to the Elastography Image Server. An advantage of this system is its high modularity, enabling various software modules to lie on the same machine or different machines. In some of the experiments two elastography image servers are run on the same computer using different GPU cards; one computing O-TRuE elastography and the other computing untracked elastography.

### Multi-Stream GPU-based Elastography

**Figure 2:**
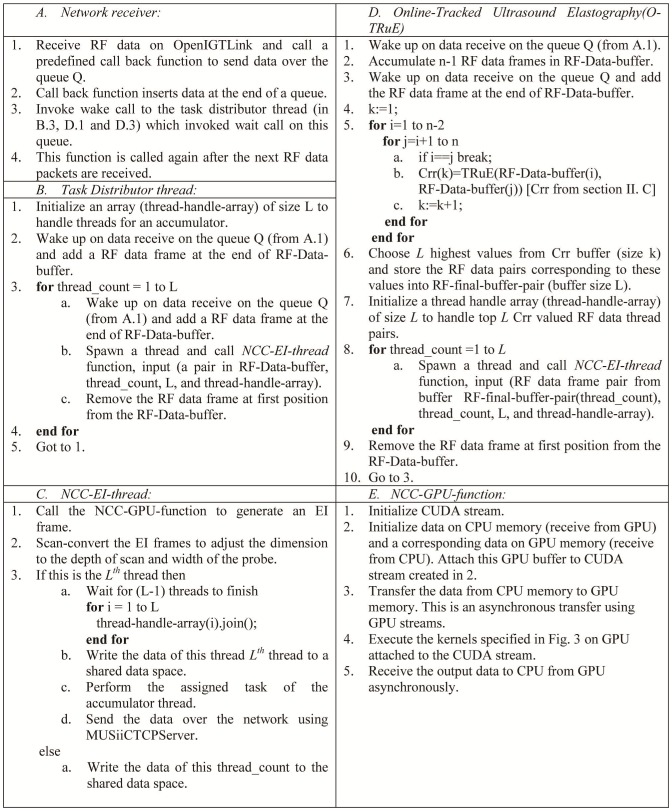
Algorithm of multi-stream GPU elastography and O-TRuE. The multi-stream GPU elastography algorithm is described on the left and the corresponding O-TRuE, which reuses several components of the multi-stream GPU elastography is on the right.

**Figure 3:**
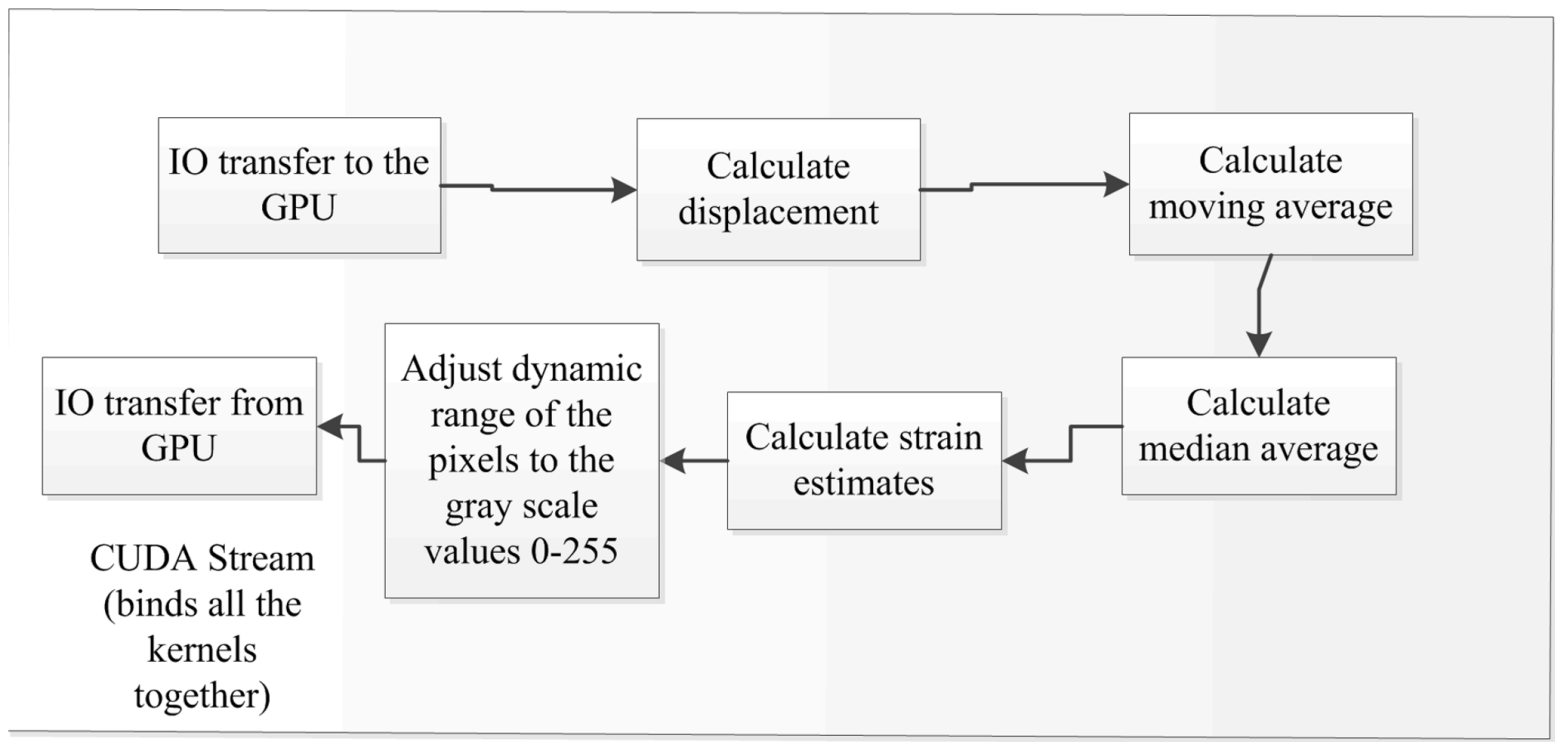
Elastography stream pipeline. Figure shows contents of the elastography image stream. These are collection of kernel calls in CUDA necessary to generate elastography images [32]. Since these streams support data integrity, they can be plugged into distinct threads.

Fig. 2 details the multi-stream GPU-based elastography algorithm. An elastography thread is a collection of normalized cross-correlation (NCC) based elastography algorithm modules as shown in Fig. 3. First, a displacement map is calculated between two RF images on GPU using NCC (eq. 1); this data is filtered using a moving average and a median filter to remove outliers from the displacement map; then strain estimation is performed using least squares fitting, followed by scan conversion. This is an extension of the work in [16] with all of these modules executing on the GPU. When multiple threads are invoked, a mechanism is needed to ensure data integrity and to prevent threads from simultaneous access to the shared data. Instead of implementing a complex mechanism of synchronizing data using indexing techniques and monitoring resource allocations, these modules are held together by a CUDA stream, which ensures data integrity within a set of CUDA kernels. Modern NVidia GPU architectures, such as Fermi, allow multiple CUDA kernels to execute in parallel with concurrent IO operations between the GPU and CPU. This high level parallelism enables optimal utilization of GPU resources.

**Figure 4:**
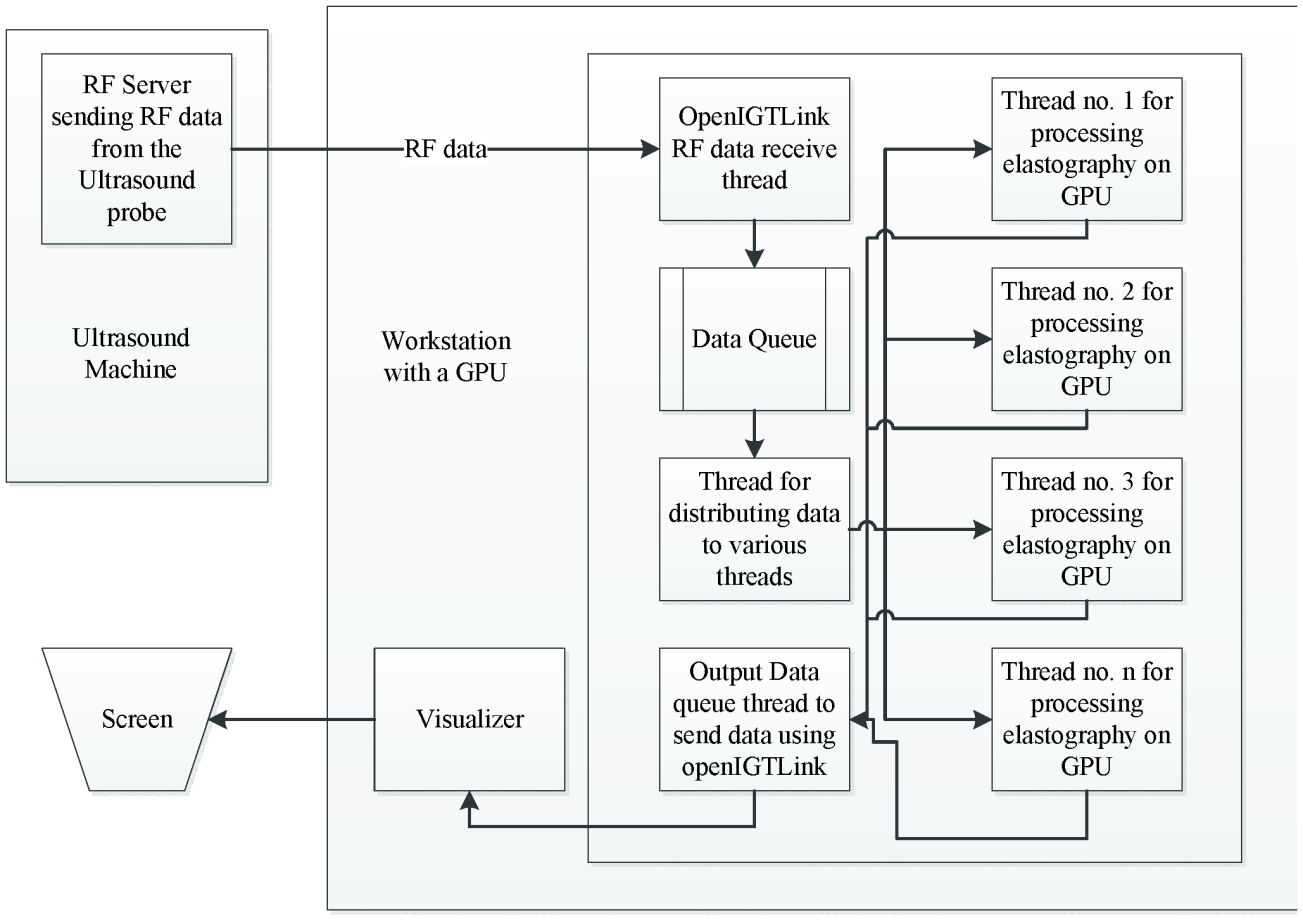
Elastography Server. This figure shows real-time pipeline where data is acquired through a radio-frequency (RF) server which runs on a US machine. As can be seen, a combination of queue and threading mechanism is implemented to connect all the components efficiently. Queuing mechanism allows the receiver and processing threads to work independently. The processing threads sleep if there is no data available to process and are triggered by data receiving component whenever data is ready. Elastography threads are the multiple threads that are spawned per consecutive or selected pair of RF data received. Every thread can send out the data over the network using IGTLMessages. The n^th^ thread can collect data from all the other n-1 threads to perform aggregate operations as averaging or weighted averaging of selective elastography images.

In our real-time ultrasound elastography system shown in Fig. 4, RF data is sent from the ultrasound machine to the elastography server. This data is then passed into a queue where the RF data is distributed over different elastography threads, each accepting a pair of RF data. Queuing mechanism helps receiver and the processing threads to work independently. The processing threads simply go to sleep when no data is available. If a data receiver thread receives data then it simply invokes a wakeup call to these threads. The Boost thread library is used for thread synchronization. The elastography threads dispatch their RF data to the GPU for elastography computation and then send the output elastography data to the output data queue of the *MUSiiCTCPServer* running an independent thread. The *MUSiiCTCPServer* may have several clients connected to it, which are typically visualizers for viewing the elastography data. To adapt this system to other usage, the n^th^ thread can simply collect *n-1* threads data to perform aggregate operations as averaging or weighted averaging of selective elastography images.

### Online Tracked Ultrasound Elastography

In Online Tracked Ultrasound Elastography (O-TRuE), a buffer of *n* RF data frames is analyzed; the Crr value from eq. (10) is calculated for these RF data frames by extracting the tracking data embedded in each RF frame. To find well-aligned RF pairs, the Crr is computed for all 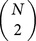 combinations of RF data frames and the top *m* matches are chosen to compute an elastography image. The algorithm is detailed in Fig. 2.

### O-TRuE Image Fusion

**Figure 5:**
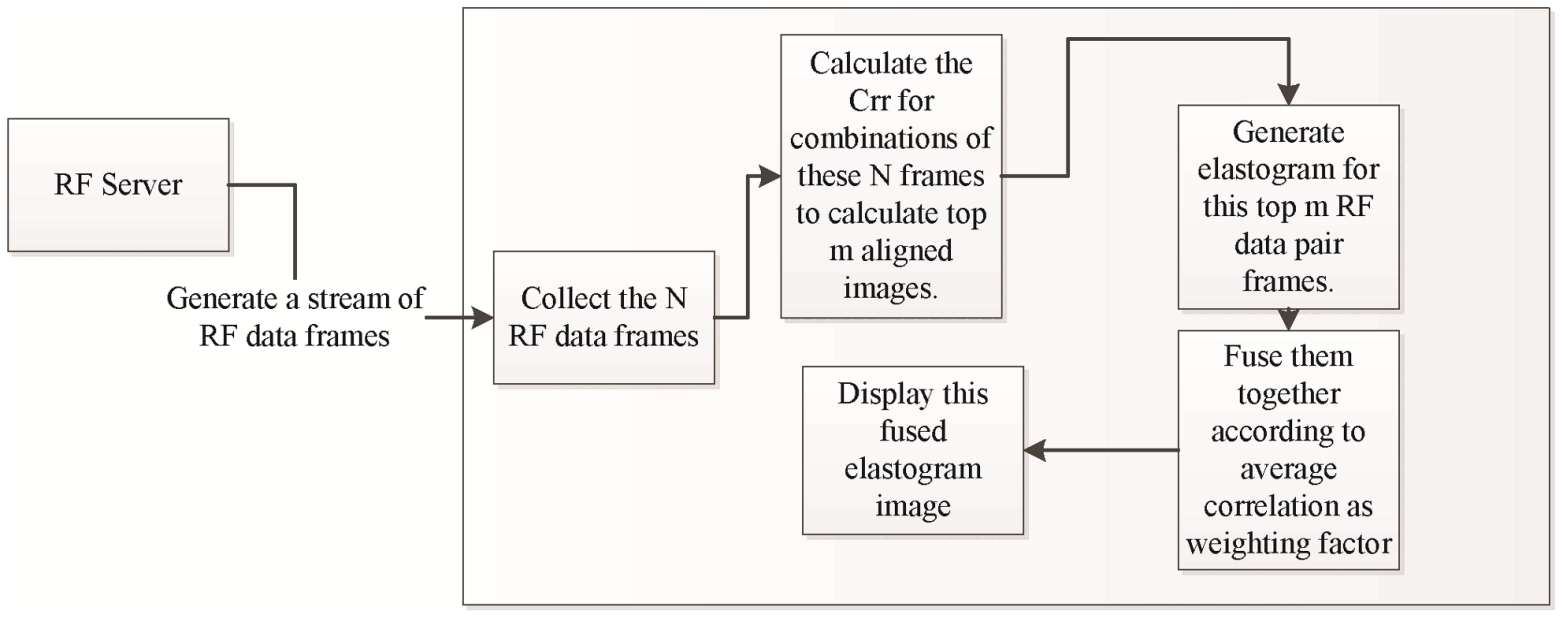
Real-time Online tracked Ultrasound Elastography (O-TRuE). Figure shows the real-time online tracked US elastography (O-TRuE) where the cost function is calculated from combinations of the tracked RF data. Then the elastography images are computed for the top *m* RF data pairs according to the Crr values. The elastography images can then be fused together by simply averaging the images or by weighted averaging based on average correlation values of each elastography image.

Image fusion of multiple elastography images may be used to compensate for global deformation, as well as improve SNR and CNR. By applying a weighted fusion, less weight may be given to the more noisy images in each fusion [10]. We investigate applying this technique to each set of *m* best matches chosen by O-TRuE as described in subsection *Online Tracked Ultrasound Elastography*. A fused image I_F_ may be defined as 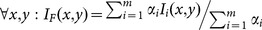
(11)where 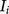 are the *m* images being fused and *α* is an image weighting factor equal to the average of the correlation map generated by NCC (Subsection *Normalized Cross-Correlation (NCC) based Elastography* within *Background* section). Fig. 5 shows the flow of O-TRuE image fusion where the top *m* strain images are fused together in real-time by weighted averaging.

We implement the image fusion operation by customizing one elastogram thread as an accumulator thread. Once the other *m-1* threads have finished calculating their elastogram images, they store these images in a shared buffer which is then accessed by the accumulator thread to compute the fused image.

## Experiments

This section details the experiments performed to show the effectiveness of the multi-stream elastography algorithm, stability of O-TRuE and stability achieved with da Vinci surgical system. Two ultrasound machines are used for the experiments, which are Sonix RP (Ultrasonix Co.) for phantom and da Vinci surgical system experiments, and Sonix CEP (Ultrasonix Co.) for in-vivo animal experiments. A high performance Tesla C2070 GPU card is used for elastography computations. The machine that is running elastography computation has 12 GB of RAM and a 2.13 GHz Intel Xeon processor.

### Phantom Experiments

In phantom experiments, a CIRS Elasticity QA Phanton Model 049, which has background elasticity of 33 kPa and lesions with 7, 15, 39, and 58 kPa elasticity, is used. The purpose of this experiments is to determine speed increment achieved by multi-stream GPU approach towards elastography, as compared to single stream and no stream GPU approach. We would like to determine whether the performance of the system in speed and quality remains stable over time on the given phantom. These results are important to establish multi-stream GPU elastography as enabling method for O-TRuE and integration with da Vinci surgical systems. In relation to this, we want to see whether O-TRuE supported freehand palpation gives any benefits over untracked freehand palpation in terms of stable correlation of consecutive images generated by both system.

The experiments are performed on the 58 kPa lesion, measuring 2 cm in diameter. The flat upper surface of this phantom helps to ensure that in-plane RF data frame detection by O-TRuE gives a very high quality elastogram. The O-TRuE algorithm consistency is measured by applying the same RF data stream as an input to both tracked and untracked version of elastography. This ensures that consistent data is used to compare the two methods. The results are saved to the disk as OpenIGTLink message files. These message files can be later retrieved for further programmatic analysis. The output elastography image is measured for consistency by measuring the correlation value of consecutive elastogram generated from O-TRuE and Untracked elastography. To compare actual sequential elastography image generation and the ones selected by O-TRuE, we save the elastography image for all permutations of the given buffer of RF data frames. We also save the information of the ranking of the elastography image frames and corresponding Crr values. The elastography frames are arranged in grid form to showcase the effectiveness of O-TRuE selection. Fusion data is also generated by combining the top *m* frames and evaluated for varying values of *m*. Typical values for *m* are 1, 3 or 5. The same RF data is used to compare different values of *m* to enable direct comparison of the results.

Multi-stream elastography algorithm speed is measured by inserting timers just before the first thread of elastography calculation is fired called 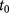 and after *N^th^* elastography thread completion. All the thread handles are collected in a dynamic array of size *N* and passed onto a separate thread along with value of 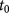. This thread waits for all *N* thread handles to indicate thread execution completion before measuring the time 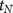; this helps to avoid delay in logging the data and prevents the elastography processing from being impacted. The time to calculate 1 elastography image frame is given by by 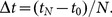
(12)


### In-Vivo Animal Experiments

The phantom experiments provide a baseline for comparison of O-TRuE and untracked elastography. The animal experiments replicate various conditions that phantom experiments cannot demonstrate. Palpation motion is not necessarily parallel to the axial motion of the probe, and the organ surface is slippery due to blood or US gel. We want to determine whether the real-time elastography implementation, due to it's high speed, compensates for the small lateral and elevational motion to give a good elastography image. A few regions of the pig liver were ablated in-vivo using RITA ablator and the ablated region was visualized using real-time untracked elastography [26]. The pig was euthanized and the liver was extracted for gross pathology study [26]. Data collection was performed by connecting a listener to the RF server. This listener saved data from the RF server in OpenIGTLink message files. The files were saved with filenames containing timestamps or sequence numbers to aid in re-playing the data at a later date. The saved RF data files were read by the untracked elastography server in the same order as they were generated. The experimental data was collected during offline TRuE evaluation as described in [26]. The depth of acquisition is 3 cm. A trend of NCC window size vs. speed of elastography image generation and image quality is conducted on the output from this in-vivo data. The SNR is calculated for the entire image, and CNR is calculated for target and background image region of (30×30 pixel square).

### da Vinci Surgical System Experiments

**Figure 6:**
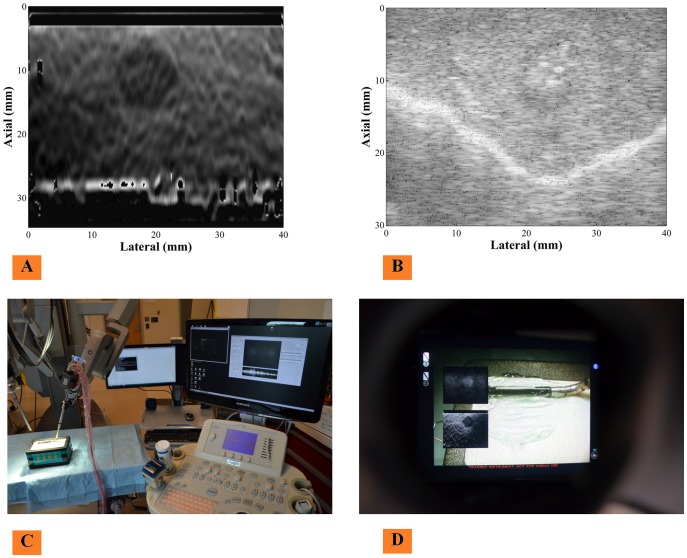
Integration with da Vinci surgical systems. Untracked elastography has been integrated with da Vinci surgical systems using a laparoscopic probe controlled by an arm of the da Vinci surgical robot. (C) Shows the overall setup. (D) Shows the view from surgeon's console of how B-mode (B) and Elastography image (A) appear when overlaid in the console display.

Under some constraints O-TRuE is hard to implement using EM tracker due to presence of ferromagnetic materials in the surrounding area. One such case is da Vinci surgical robot where the US probe is mounted on one of the arms of the robot [27]. We demonstrate the feasibility of integration of elastography with a da Vinci surgical robot where controlled palpation motions are performed. We want to determine what type of palpation motion can give a steady elastography image stream and if high speed elastography has any advantage. As shown in Fig. 6, the da Vinci surgical robot is connected with the US machine using OpenIGLTLinkMUSiiC where the B-mode and it's corresponding elastography images are sent over the network. The console of the da Vinci surgical robot has live frames of elastography and B-mode overlaid within surgeon's field-of-view; the enabled status, position and size of these frames can be adjusted using the master manipulator of the robot to provide the surgeon full control. Palpation motion is generated by the robotic arm of the da Vinci using the da Vinci research API to autonomously control the frequency, amplitude, and direction of robot motion [27].

## Results

### Speed Analysis

Results of a run-time performance comparison of multi-stream GPU elastography with single-stream and non-stream GPU elastography is provided in Table 1. Frame rate averages and standard deviations are computed for each GPU implementation under different test cases of varying number of RF lines, NCC window size, NCC maximum search distance, and NCC search step size. As seen in Table 1, the runtime differences between non-stream and single-stream are negligible. This indicates low overhead in our implementation of streamed data processing. For the multi-stream implementation, the runtime speedup is very significant and in some cases more than double the frame rate of the other implementations. In some cases, the multi-stream implementation with 256 RF lines is even faster than the normal and single-stream implementations with 128 RF lines. This indicates that our multi-streamed implementation provides higher utilization of the GPU with greater runtime efficiency, even though the multi-stream implementation is controlled from multiple CPU threads. As seen in the Table 1, the highest speed achieved is 78 fps while running the multi-stream implementation with 128 RF lines. This is a significant improvement given that the corresponding rate of elasticity image generation nearly matches the image acquisition speed of the RF server as presented in [31].

**Table 1:** Test results for comparing frame rate performance of multi-stream GPU elastography (threaded) with single-stream (streamed) and non-stream (normal) GPU elastography.

		Depth in cm	4	5	6	7	8	9
		Depth in pixels	1024	1296	1552	1808	2064	2336
Case	1	normal-128	36.56(±1.75)	31.10(±0.61)	31.56(±0.29)	29.10(±0.39)	25.20(±0.20)	22.39(±0.28)
Window size (pixel)	10	threaded-128	**78.02(±0.92)**	61.14(±0.53)	48.40(±0.87)	46.19(±0.48)	39.36(±0.58)	34.33(±0.34)
Displacement (mm)	2	streamed-128	36.31(±2.22)	29.01(±6.02)	30.76(±0.97)	29.41(±0.34)	25.19(±0.47)	22.24(±0.11)
Overlap (%)	98	normal-256	26.10(±0.16)	22.72(±0.41)	19.08(±0.06)	17.80(±0.07)	15.66(±0.32)	13.89(±0.03)
		threaded-256	**42.55(±0.96)**	31.48(±0.41)	26.78(±0.63)	24.23(±0.33)	21.78(±0.36)	19.60(±0.09)
		streamed-256	26.00(±2.49)	22.60(±0.38)	19.13(±0.09)	17.77(±0.19)	15.27(±1.38)	13.75(±0.21)
Case	2	normal-128	34.74(±1.65)	31.98(±0.32)	30.62(±0.68)	27.71(±0.21)	24.11(±0.26)	21.03(±0.12)
Window size (pixel)	12	threaded-128	**72.07(±1.54)**	56.43(±0.89)	45.55(±0.70)	42.42(±0.57)	36.72(±0.27)	32.41(±0.22)
Displacement (mm)	2	streamed-128	34.68(±3.02)	31.91(±0.50)	30.56(±1.15)	28.29(±0.79)	24.49(±0.46)	21.20(±0.22)
Overlap (%)	98	normal-256	24.36(±0.75)	20.52(±0.41)	17.94(±0.07)	16.34(±0.33)	14.64(±0.19)	12.77(±0.03)
		threaded-256	**39.04(±1.34)**	28.54(±0.80)	25.55(±0.27)	22.35(±0.44)	20.59(±0.26)	18.27(±0.07)
		streamed-256	24.16(±2.20)	21.21(±1.57)	18.08(±0.43)	16.53(±0.33)	14.82(±0.05)	12.78(±0.07)
Case	3	normal-128	21.53(±0.13)	21.54(±0.12)	19.73(±0.08)	19.19(±0.03)	16.98(±0.12)	14.24(±0.04)
Window size (pixel)	14	threaded-128	**46.43(±0.44)**	35.44(±0.42)	29.53(±0.29)	27.05(±0.20)	24.46(±0.35)	21.30(±0.07)
Displacement (mm)	4	streamed-128	21.76(±0.13)	21.60(±0.13)	19.84(±0.17)	19.33(±0.11)	16.63(±0.25)	14.35(±0.09)
Overlap (%)	98	normal-256	15.74(±0.07)	13.67(±0.03)	11.56(±0.15)	10.69(±0.03)	9.67(±0.04)	8.12(±0.02)
		threaded-256	**25.89(±0.19)**	19.53(±0.15)	16.62(±0.12)	14.47(±0.21)	13.37(±0.12)	11.67(±0.06)
		streamed-256	15.83(±0.09)	13.68(±0.13)	11.50(±0.04)	10.68(±0.06)	9.68(±0.02)	8.12(±0.01)
Case	4	normal-128	19.71(±0.09)	16.45(±0.13)	13.65(±0.05)	12.49(±0.08)	11.15(±0.05)	9.71(±0.04)
Window size (pixel)	16	threaded-128	**28.93(±0.24)**	25.23(±0.68)	20.65(±0.08)	17.73(±0.24)	15.11(±0.09)	14.01(±0.19)
Displacement (mm)	4	streamed-128	19.81(±0.10)	16.24(±0.50)	13.66(±0.19)	12.48(±0.18)	11.12(±0.13)	9.69(±0.04)
Overlap (%)	99	normal-256	11.64(±0.02)	9.17(±0.02)	7.64(±0.02)	6.82(±0.06)	6.08(±0.02)	5.20(±0.01)
		threaded-256	**15.70(±0.07)**	13.02(±0.07)	10.21(±0.11)	9.00(±0.13)	8.10(±0.02)	6.64(±0.04)
		streamed-256	11.57(±0.31)	9.20(±0.06)	7.65(±0.05)	6.80(±0.08)	6.02(±0.11)	5.19(±0.02)

This table reports average frames per second (with standard deviation in brackets) of images generated by various versions of the elastography program. The term *normal-N* indicates the basic GPU implementation of NCC elastography, *streamed-N* indicates the streamed GPU implementation, and *threaded-N* indicates the multi-streamed GPU implementation, where *N* indicates the number of RF lines in each RF image. Four test cases were performed at different NCC window sizes, NCC maximum search distances (displacements), and NCC search step sizes (specified as percentage of window overlap). The computational load increases with larger window size, displacement, and percent overlap. As seen in the results, the highest speed obtained is 78 frames per second (fps) running the multi-streamed GPU implementation.

**Figure 7:**
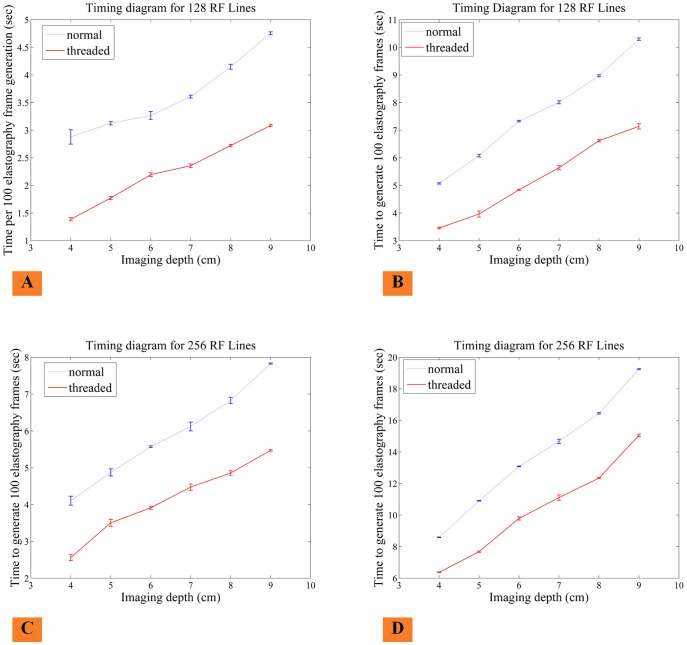
Timing graph to show speed comparison of multi-stream elastography (threaded) and non-stream elastography (normal). The graphs indicates run times and standard deviation of run time for window size 12, displacement 2 mm, overlap 98% (A, B) and Window size 16, displacement 4 mm, overlap 99% (C, D). The results are per 100 frames. The standard deviation is max 0.13 for Fig. (A), 0.122 for Fig. (B), 0.136 for Fig. (C), 0.167 for Fig. (D), which is very small for 100 frames. This graph also shows that the increased window size reduces the performance of the algorithm due to higher serial search within the large windows.

Fig. 7 provides a graph of inverse results of a subset of Table 1, which shows the average generation time in seconds for 100 elastography frames estimated over 20 trials. Fig. 7 compares non-stream and multi-stream GPU implemenations, making clear that multi-stream outperforms non-stream. The bars in the figure indicate the standard deviation of runtime among the 20 trials. A stable runtime is important to ensure fast system response over all periods in time. Fig. 7 shows that the standard deviation for both GPU implementations is stable, the standard deviation is max 0.13 for Fig. 7-A, 0.122 for Fig. 7-B, 0.136 for Fig. 7-C, 0.167 for Fig. 7-D. A worst case standard deviation of 0.167 seconds to generate 100 elastography frames (Fig. 7-D) indicates a stable runtime.

### Validation of O-TRuE Frame Selection

**Figure 8:**
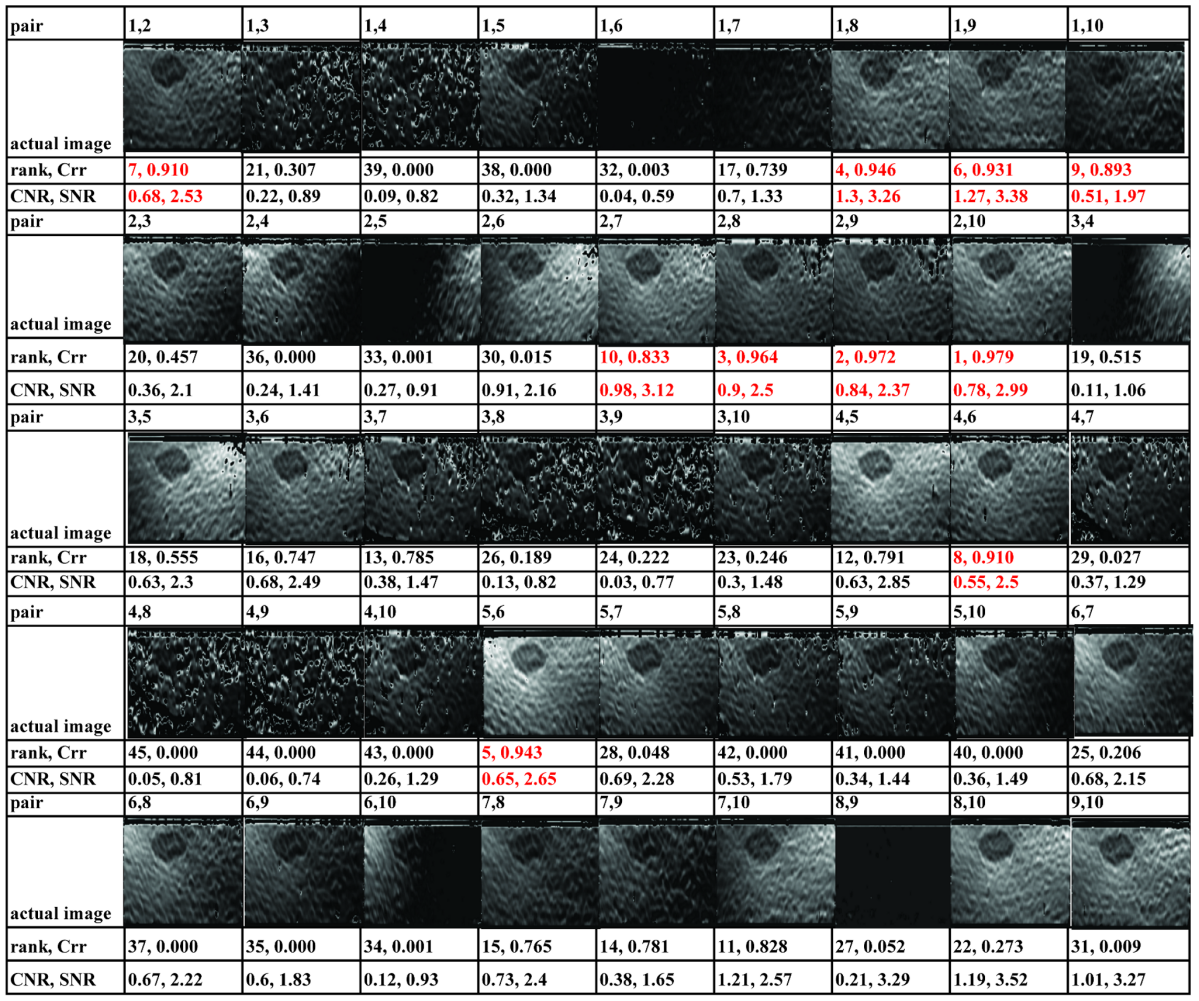
Selection map of O-TRuE images. The row above each image sequence indicates the RF data pair index. For e.g. the pair identifier (n1, m1) indicates comparison of radio frequency (RF) data frame acquired at time 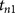 with that of the frame acquired at time 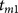. The pair (image rank, Crr value) below the image sequence indicates the rank and Crr value generated by O-TRuE. The pair (CNR, SNR) indicates contrast-to-noise ratio and signal-to-noise ratio values for each image. O-TRuE selected 90% good elastography images in top 20 ranked images with good CNR and SNR above 0.51 and 2.37 respectively. The Crr above 0.457 is observed to provide with good elastography images.

A validation of O-TRuE is performed by computing a Crr pseudo correlation value (eq. 10) and a corresponding elastography image for all possible 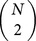 RF frame pairs in an N sized buffer with N equal to 10 and σ equal to 1 (see eq. 2 and 7). In a non-validation context, only the frames with highest Crr values would have been chosen for computing corresponding elastography images. Fig. 8 presents the generated elastography images from this test, which are arranged by order of RF frame acquisition. Visual inspection reveals that 90% of the top 20 frames chosen by O-TRuE show clear presence of the lesion being imaged. As a quantitative assessment, the CNR and SNR values of each elastography image is calculated and listed as a pair (CNR, SNR) below each image. It is found that top ranking elastograms have either a very good CNR or a very good SNR value, whereas the O-TRuE images of lower rank (i.e. lower Crr) have poorer values of CNR and SNR. For example, the image with rank 26 has a very low CNR of 0.13 and SNR of 0.82. It is observed that the 10 highest ranking O-TRuE images (shown as red text in the figure) all have CNR above 0.51 and SNR above 2.37, which indicates a good elastography result. From these tests, we observe that choosing elastography images with Crr values above 0.457 provides a mostly stable result. There are few anamolies, such as the image with rank 12 having better image quality than the image of rank 9. Such anomalies could be corrected by considering the CNR and SNR values in the ranking system, but at the cost of reduced speed due to the added burden of generating additional elastogram images in order to compute the CNR and SNR values across an extended range of Crr ranked RF frame pairs. In general, these test results show that O-TRuE performs very well in selecting the best RF frame pairs to generate high quality elastograms.

### O-TRuE Image Fusion Evaluation

**Figure 9:**
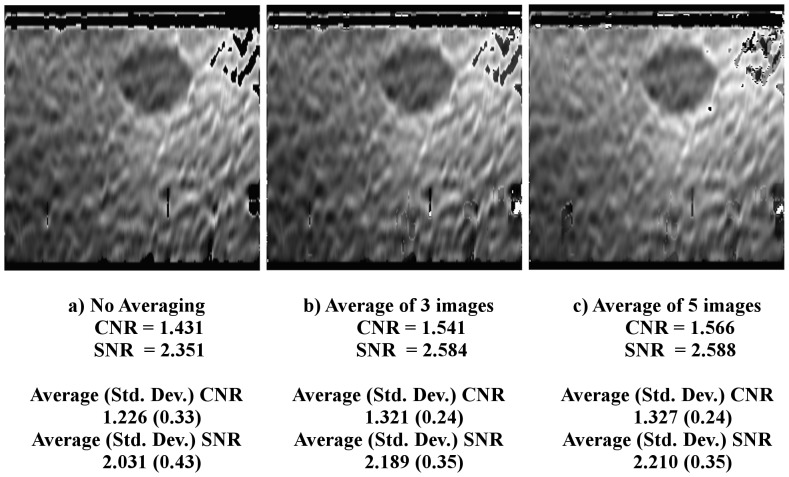
Elastography image fusion. The images displayed in (a) is elastography image with single image (best O-TRuE) selection, (b) is elastography image for average of top 3 O-TRuE image selections, and (c) is elastography image for average of top 5 O-TRuE image selections. The results indicates that the fusion by averaging the top 5 elastography images from O-TRuE gives good quality indicated by the average CNR and SNR values of 1.327 and 2.210 respectively.

An analysis of the effects of image fusion by averaging is presented in Fig. 9 for elastography images generated by O-TRuE. As seen in Fig. 9, (a) represents the O-TRuE output for single elastography image of highest Crr value with no averaging, (b) represents the O-TRuE output when averaging the top 3 images of highest Crr value, and (c) represents the O-TRuE output when averaging the top 5 images. Fig. 9 shows the CNR and SNR values of each image, as well as average CNR and SNR values evaluated from approximately 220 output images for each case, indicating that fusion by averaging of 5 images provides the best CNR and SNR with average values of 1.327 and 2.210 respectively. This indicates that SNR and CNR performance improves by averaging, but it may fail in cases where some of the top images are noisy as suggested by Fig. 8 where images ranked 2 and 3 contain noise in the top right corner of the images. This test indicates that averaging of 5 O-TRuE images is useful for performing image fusion.

### Elastography Image Stream Analysis

**Figure 10:**
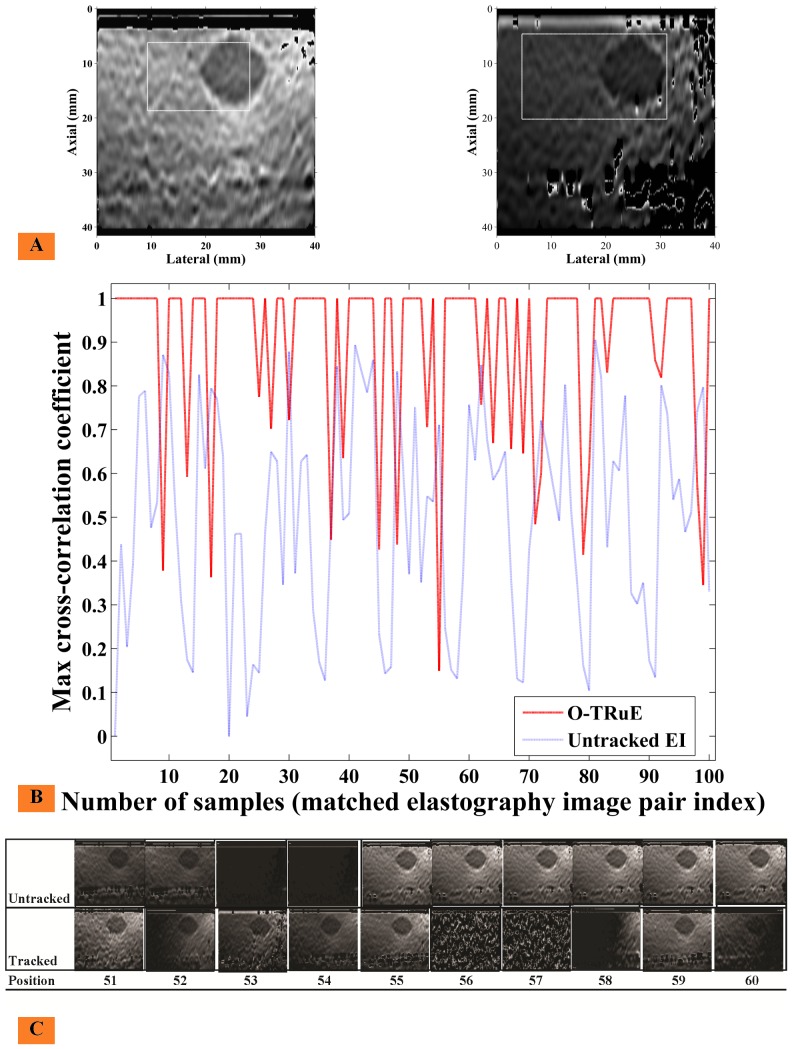
Elastography image stream analysis of consecutive frames in O-TRuE and Untracked elastography. An analysis of consecutive frames is done to understand the quality of strain images generated by O-TRuE and untracked elastography. (A) Shows a template region selected in the leftmost image and a target region selected in the rightmost image. We apply normalized cross-correlation in these regions as shown in eq. 13 to find max correlation value. A max correlation graph for 100 elastography image pairs is shown in (B), where the red dashed line is for O-TRuE and a blue dotted line is for untracked elastography. O-TRuE has a more consistent high correlation value across consecutive images. As indicated in Table 2, O-TRuE (β values) performs better than untracked elastography. (C) Shows the dataset for frames in range [51, 60]; here O-TRuE has its lowest cross-correlation value from 53 to 54; as can be seen, the image quality drastically changes in this range.

A consistency analysis is performed for the elastography image streams of both O-TRuE and untracked elastography by applying NCC to a sub-region of the output elastography images as shown in Fig. 10-A, where the left-hand image shows the defined size and position of the NCC template window for a given elastography image and the right-hand image shows the defined search region for the target window in the subsequent elastography image. Similar to eq. (1), a correlation map is generated within the target search region and the maximum correlation value is selected from the map as the stream quality measurement
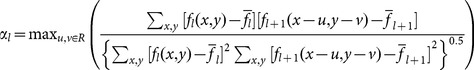
(13)where *l* is an output elastography image sequence number with value from 1 to the number of RF image pairs minus one, x (lateral) and y (axial) are pixel positions within the template window 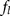 in image *l*, (x-u) and (y-v) are pixel positions within a target search window 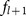 in image (*l+*1). Note that 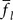 and 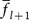
are the mean pixel intensities of the 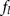 and 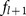 window regions respectively. The maximum correlation value of the map corresponds to the position of optimal alignment between image features contained by the template and target windows. Thus, the maximum correlation value is the primary value of interest within the map and is sufficient to serve as an indicator of stability for the image stream.

The region of interest (ROI) in Fig. 10-A was manually selected following data acqusition in such a way to contain the lesion being imaged while being large enough to accommodate small displacements of the lesion due to hand motion. This enables running the NCC analysis on a continuous stream of data while ensuring that the lesion remains within the imaged area. A larger window size would risk inclusion of noise along the boundaries of the elastography image, so the window size and position is appropriately defined for the image target in this study. The window size and position is kept constant for both O-TRuE and untracked elastography image streams.

Fig. 10-C shows a subset of the sample elastography image stream with corresponding quality measurements for the full stream sample in Fig. 10-B where consecutive elastography images are compared using NCC. When comparing the max correlation of consecutive frames obtained from O-TRuE and normal (untracked) elastography, it is found from the graph in Fig. 10-B that O-TRuE has high and relatively stable correlation, whereas untracked elastography has correlations of low and more rapidly varying values.

We represent the percentage of images having a correlation value above a user-defined threshold 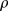 for a given value of 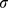
(eq. 2, 7, and 10) as
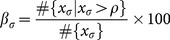
(14)where 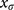 is an array of correlation values for an image stream acquired using the setting σ, 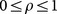
is the threshold on the correlation values, and ‘#’ is the standard set notation indicating the number of elements in a set. Table 2 shows the percentage of frames having max correlation values above 0.6 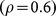
for different O-TRuE buffer sizes and for with step size 1 (see eq. 2, 7, and 10). Fig. 10 shows results for 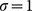
and buffer size 10. The last column in the table provides results for normal/untracked elastography. Table 2 indicates that O-TRuE outperforms untracked elastography at all buffer sizes by a factor of ∼1.9X to ∼2.7X. It is thus observed that O-TRuE is more stable than untracked elastography in terms of consistent image quality. A snapshot of an elastography image frame sequence for frames 51–60 is shown in Fig. 10-C. Due to the moving window buffer, O-TRuE sometimes picks up the same RF frame pair as the previous image in the stream sequence. It can be observed that the frame correlation for O-TRuE drops dramatically from 52 to 53. There is a rise between frames 53 and 54 because the images are constant although void of features. The correlation drops again from frame 54 to 55 but is stable for the remainder of the subset. On the other hand, for the untracked image stream correlation is low for the majority of frames.

**Table 2:** Percentage of consecutive frame pairs above a certain threshold of max correlation for varying σ values as described in eq. 2, 7, 10 and eq. 14.

Buffer size	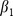	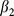	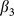	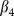	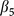	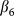	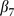	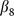	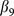	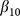	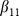	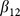	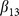	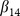	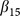	Untracked elastography
10	91.07	90.05	88.78	86.99	86.73	84.69	83.42	84.18	82.14	83.93	84.69	84.18	84.18	84.18	84.69	43.11
20	95.26	93.95	93.79	93.79	92.81	91.18	90.85	89.54	88.56	88.24	87.91	86.93	85.29	86.27	85.95	36.60
30	94.95	95.90	92.74	92.74	93.69	93.38	92.74	91.80	91.17	90.85	92.11	93.38	92.11	92.11	91.48	33.44
40	97.11	96.06	96.33	97.11	95.80	95.28	94.23	95.54	95.28	93.96	92.91	92.65	92.91	93.18	92.13	41.73
50	98.66	98.39	98.12	97.31	96.77	95.97	94.09	94.35	94.89	94.89	94.09	93.28	92.74	93.82	93.28	37.37

As an example 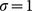 (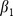) and buffer size 10 indicates percent of correlation values above the range 0.6 for the graph in Fig. 10. As can be seen in this table, in most cases, the quality of the output system improves with the increasing buffer size.

### In-Vivo Animal Experiments

**Figure 11:**
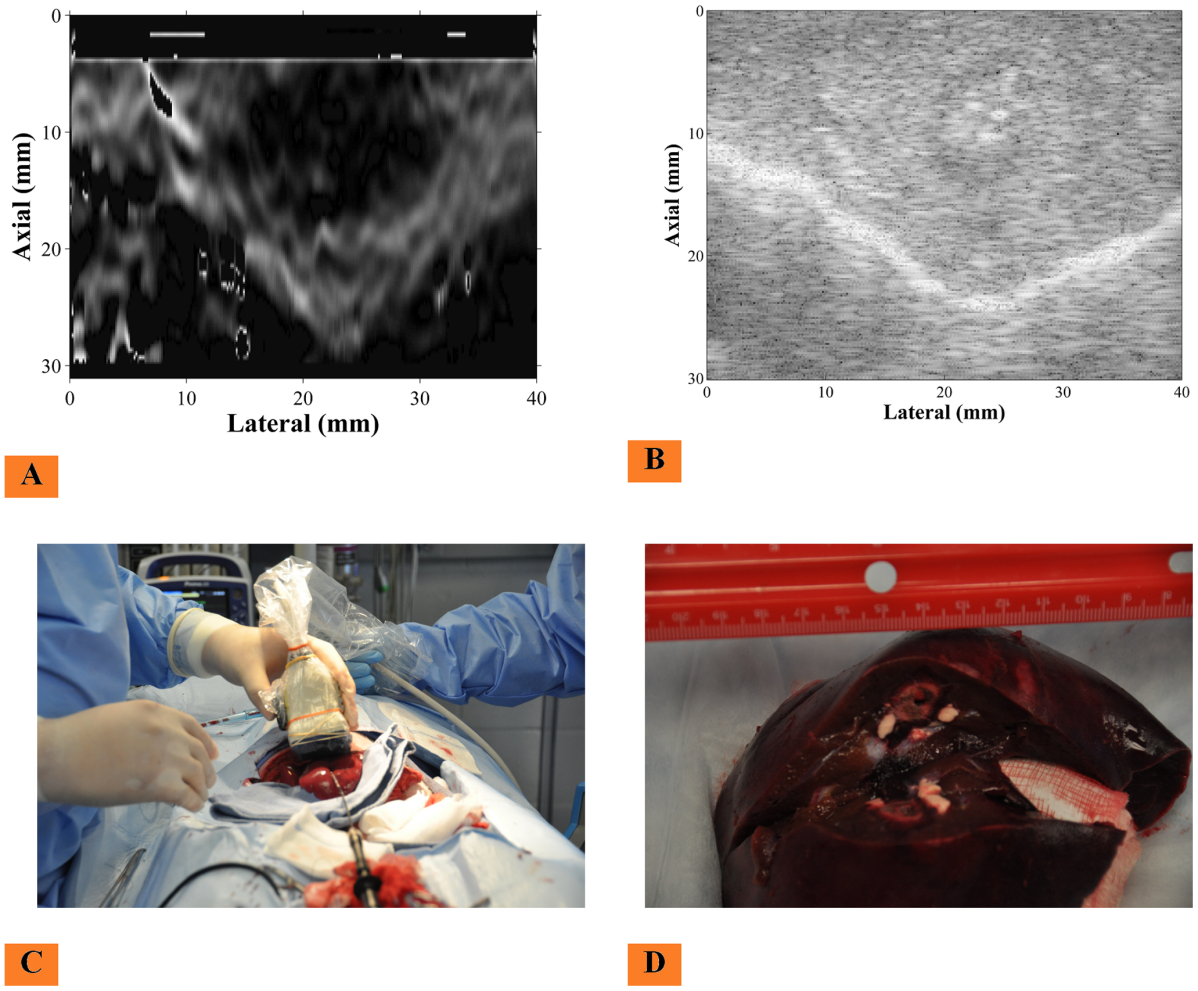
Animal Experiment setup. An in-vivo animal experiment was performed on a pig liver; an ablation was induced in the liver using RITA ablator as shown in (C). Elasticity image can be seen in (A), corresponding B-mode image in (B). The ablation region was approximately 2 cm in diameter as validated by gross pathology of the liver in (D).

Here we present results from the in-vivo animal ablation study described in subsection *In-Vivo Animal Experiments* within *Experiments* section. As seen in Fig. 11-C, a 4DL14-5/38 probe was placed just above the ablation region of the liver for collecting 2D data. Fig. 11-A shows the elastography image of the ablated region. As can be seen, it has a better contrast than the corresponding B-mode image in Fig. 11-B. Fig. 11-D shows the gross pathology of the ablated region, which shows an ablation of approximately 2 cm in diameter. In Fig. 11-B the contour of the ablated region is not clearly visible since the thermal transfer did not drastically change the acoustic impedance of the tissue. Elastography images as shown in Fig. 11-A clearly shows a better contrast and boundary of the ablated region. This indicates that the multi-stream GPU-based elastography functions well in in-vivo experiments.

**Figure 12:**
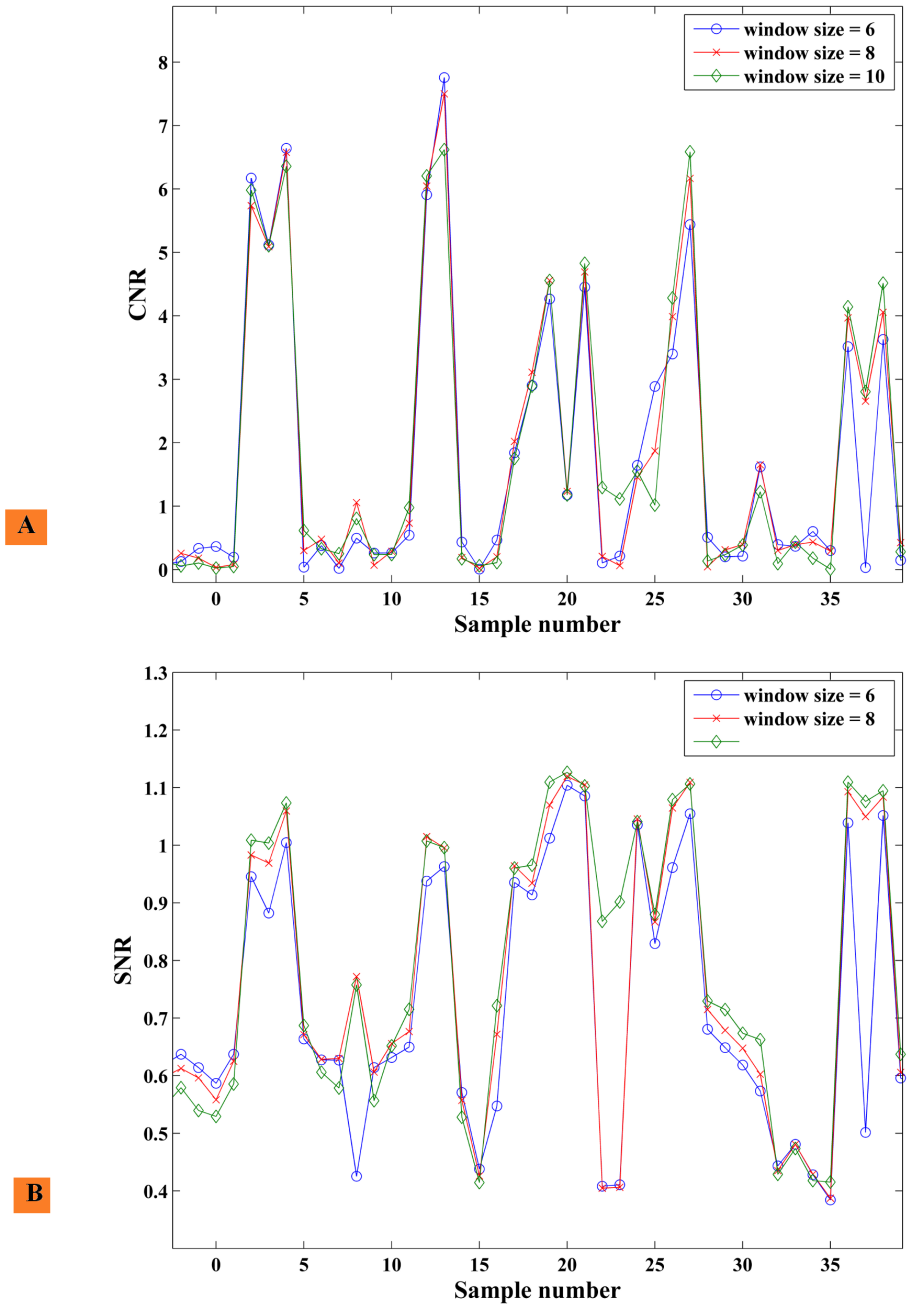
Trend of untracked elastography for in-vivo pig data: NCC window size vs. CNR and SNR. The graph shows variation of CNR and SNR of individual sample points for different NCC window sizes with untracked elastography. The data was obtained from in-vivo experiments on 350 samples and 199 samples were selected after ignoring invalid strain values. (A) Shows snapshot of CNR values and (B) shows snapshot of SNR values varying for a small subset of the 199 samples. The average/min/max values of the CNR and SNR are listed in Table 3. The CNR and SNR across different window sizes are closely related per sample but the global variation in CNR and SNR is high due to wide range of values.

CNR and SNR for the elastography images were calculated at NCC window sizes of 6, 8, 10, 12, 14, and 16 using a fixed maximum NCC search distance of 2 mm and step-size overlap of 98% on 350 images. Around 200 images were chosen after ignoring de-correlated RF image pairs due to the effect of out-of-plane motion. Elastography computation speed was assessed by processing the first 100 RF image pairs 20 times. The effect of varying window size on speed and on mean, max, and min CNR and SNR values is presented in Table 3. It can be seen that optimal mean CNR (3.56) and near-optimal mean SNR (0.94) is achieved for window size 10. The SNR value increases as we increase the window size; this happens because increasing the window size while keeping the percentage of overlap the same results in cross-correlation being computed on a bigger area to find the best match between the template and target areas in two images. CNR initially increases with window size but decreases moving beyond window size 10. There is a wide range of CNR values observed in the images, as evidenced by the high standard deviation and min/max values at each window size. A closer look at this variation is provided in Fig. 12, which plots the CNR and SNR computed for each sample image at the first three window sizes.

**Table 3:** Untracked elastography: NCC window size vs. speed and image quality.

		CNR		SNR	
window size	fps	min/max	mean	min/max	mean
6	56.23(±0.71)	0.0027/9.05	2.92(±2.31)	0.3572/1.43	0.82(±0.25)
8	52.07(±0.96)	0.0054/9.05	3.41(±2.44)	0.3782/1.43	0.90(±0.24)
10	48.16(±0.57)	0.0075/9.18	3.57(±2.41)	0.4128/1.39	0.94(±0.21)
12	44.87(±0.57)	0.0091/9.20	3.53(±2.37)	0.4140/1.37	0.95(±0.20)
14	41.68(±2.47)	0.0396/8.76	3.40(±2.21)	0.3993/1.36	0.97(±0.19)
16	39.65(±0.66)	0.0673/8.33	3.28(±2.11)	0.3455/1.28	0.97(±0.18)

The table shows the change in frame rate and in CNR and SNR according to NCC window size of the multi-stream elastography. We varied the window size while fixing the maximum NCC search distance at 2 mm and overlap of 98%. The CNR and SNR were averaged for 198 images. The speed was calculated by calculating elastography images for the first 100 RF pairs 20 times. It is found that window size of 10 is optimal with high mean CNR and a good mean SNR value; although the highest mean SNR value corresponds to window size 14. This table indicates that as the window size increases the mean CNR and SNR increase along with a reduction in frame rate. Intermediate frame rates corresponding to window sizes 8 and 10 give satisfactory mean CNR and SNR and a high frame rate of 52.07 and 48.16 respectively.

### da Vinci Surgical Robot Palpation Analysis

**Figure 13:**
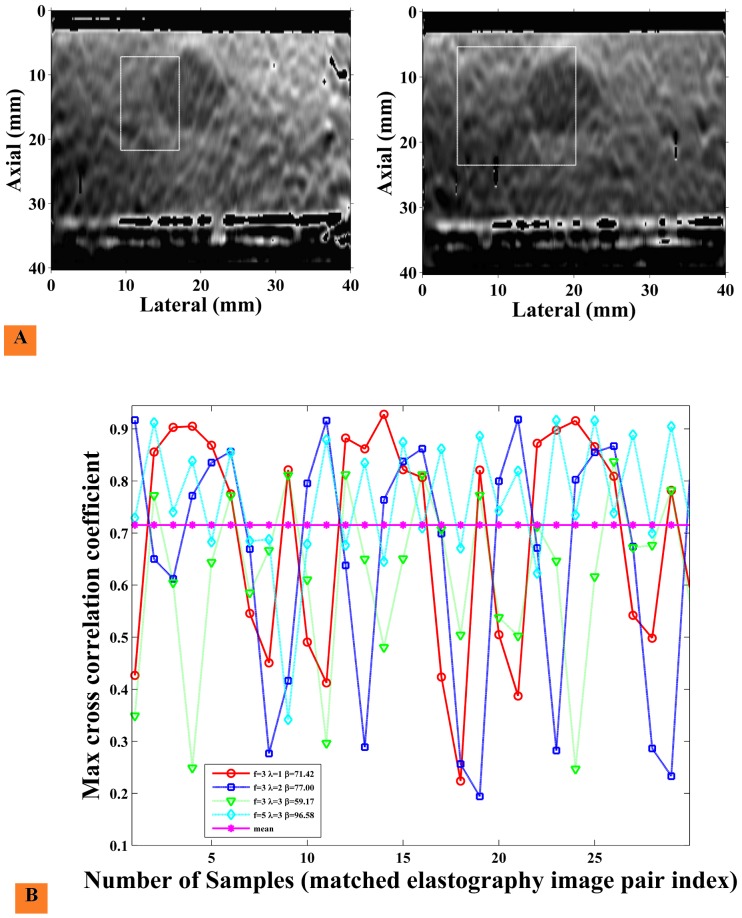
Max cross-correlation graph of consecutive images from robot assisted palpation. Max cross-correlation graph performed on consecutive frames for da Vinci surgical system. (A) Shows a template region selected in the leftmost image and a target region selection in the rightmost image. We apply normalized cross-correlation in these regions as shown in eq. 13 to find max correlation value. (B) Shows a mean correlation graph of initial 30 elastography image pairs out of 1200 elastography image pairs. Palpation parameters is expressed as frequency (*f*) in Hz and amplitude *λ* in mm. High correlation indicates a good match in consecutive frames; clearly,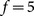, 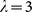 indicates a very stable and consistent result with 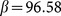, where 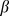 is defined in eq. 14. Each β value is calculated for 1200 elastography image pairs.

We apply elastography stream analysis on untracked elastography images generated by robot assisted palpation using the da Vinci Surgical System. The normalized cross-correlation between matched template and target regions of sequential output elastography images for different palpation frequencies and commanded amplitudes is shown in Fig. 13-B. Fig. 13-A shows the NCC template region and the NCC target search region applied to the output elastography images. At a frame rate of 20 fps with a laparoscopic ultrasound (LUS) probe, it is observed that a very high β value (as defined in eq.14) of 96.58 is obtained corresponding to the palpation frequency of 5 Hz and commanded amplitude of 3 mm giving the most stable elastography stream. Each β value is calculated for 1200 elastography image pairs. The graph in Fig. 13 follows a sinusoidal pattern; this pattern reflects the sinusoidal motion of the LUS probe attached to the arm of the da Vinci system. This sinusoidal motion is reflected in all the cases presented in Fig. 13. The results of the remaining combinations of frequency and amplitude are above 0.6. These results quantify the quality of the output elastography stream for robot controlled elastography.

## Discussion

This paper presented an exhaustive study of a real-time multi-stream GPU-based elastography system with demonstration in three applications including tracked (O-TRuE) phantom experiments, untracked in-vivo experiments, and untracked phantom experiments with robot controlled palpation. The real challenge is to tune the implementation of the complex elastography algorithm to meet the needs of a practical real-time system, i.e. it has to be reliable, have constant response time, and provide high-quality results. In addition, our system is highly modular and cost effective due to increasing performance of main-stream GPGPUs.

The maximum speed of 78 frames per second achieved by our elastography system approaches the RF data acquisition speed of current ultrasound systems. These results were obtained by calculating elastography over the entire image. Further performance improvement could be achieved by limiting the elastography computation to a sub-region of the image once it has been established that a target of interest, such as a tumor, is located in a particular area of the image. Initial improvement can be achieved by ignoring the border samples of RF data since tissue compression due to transducer motion is more axial in the central area of the image.

The system is highly modular and connected via the OpenIGTLinkMUSiiC API. This grants the ability to connect our system to various open source frontend modules, such as 3D Slicer for advanced visualization of the image stream. Since tracking information is embedded in each frame, advanced visualizers could, for example, allow spatial visualization of the elastography data in correspondence with 3D B-mode data. This feature also enables the ability to store and retrieve elastography data based on tracked position and timestamp. The highly modular framework enables the algorithm to run on multiple GPU's stationed at one or multiple computers and to combine streams of data from various sources. Data synchronization and ordered sequencing from multiple GPU's can be a challenge, but it is achievable.

Our current system suffers from some network latency between the different system components, including the ultrasound machine, GPU server, and data synchronizer. One potential solution is to integrate the system onto one ultrasound machine and connect various components through memory mapped inter-process communication. A clear advantage of our current implementation, however, is that the CPU of the ultrasound machine is not tasked beyond its primary function of RF data acquisition.

The ranking of O-TRuE images in Fig. 8 show that a Crr value above 0.45 provides a good quality elastography image. The relatively stable correlation values shown in Fig. 10 for images generated by O-TRuE indicates that the O-TRuE algorithm increases the stability of the output image stream over untracked data. A possible optimization of the algorithm to further stabilize the output image stream would be to filter out frames having poorly correlated RF image pairs by collecting empirical evidence to establish a lower threshold on the Crr value required to produce a good elastography result. In addition to transducer motion, movement of the patient may also affect the quality of an elastography image. Since patient motion is untracked, O-TRuE cannot currently account for this. An image based tracking mechanism could be used as an adjunct to detect patient motion, such as by applying NCC to a small region in the center of the RF data to compute image motion in both lateral and axial directions. This information could then be used by O-TRuE when computing Crr values in order to make the algorithm more robust to patient motion. The O-TRuE algorithm could also be applied in the context of robot assisted palpation using the robot's kinematics to track the position of the ultrasound probe.

Image fusion of multiple elastography images helps to improve the quality of the elastography image, but can potentially add noise to the image. Possible approaches to address this could be enforcing a minimum threshold on the Crr value as discussed earlier and increasing the buffer size with the support of multiple GPUs if needed.

As indicated by our assessment of robot assisted palpation in Fig. 13, the stability and quality of the output elastography image stream is affected by variation of the palpation frequency and displacement. An enhancement to the system would be to use the measured image correlation as feedback to the robot to autonomously vary the frequency and amplitude of palpation to determine the optimal setting. A high speed elastography engine as we have presented is a necessary prerequisite to enable such an approach.

The in-vivo animal experiment showed good contrast between the ablated region and background tissue. Table 3 and Fig. 12 indicate that high speed untracked elastography provides good quality CNR and SNR values. A more exhaustive study would help to more fully understand the effects of window size on speed, CNR and SNR.

## Conclusions

This paper presents a multi-stream GPU-based implementation of elastography, specifically demonstrating how recent advancements in GPU hardware may be harnessed to achieve much higher frame rates than previously possible. Our system achieves approximately 2.13X improvement over a conventional GPU-based NCC elastography implementation and produces a maximum frame rate of 78 fps, nearly matching the acquisition rate of typical ultrasound systems.

We demonstrated the versatility of our architecture by implementing an online version of tracked ultrasound elastography (O-TRuE), by in-vivo animal experiments, and by integration with the da Vinci Surgical System. We devised a method to evaluate the quality of an output elastography stream based on the maximum correlation of a windowed region defined in consecutive elastography frames. Using this metric, we demonstrated that O-TRuE (with tracked free-hand elastography) produces a more stable output stream than untracked free-hand elastography. A comparative study was performed to assess the effect of NCC window size on elastography frame rate and image quality. With in-vivo pig data, the optimal NCC window size of 10 provided a speed of 48 fps with CNR of 3.57 and SNR of 0.94. The in-vivo animal experiment using untracked elastography demonstrated better contrast of ablated regions in the elastography images compared to the corresponding B-mode US images. Integration with the da Vinci system investigated the effect of palpation frequency and amplitude on elastography image quality with an elastography phantom, finding a stable output stream using 5 Hz and 3 mm, respectively. These experiments demonstrate the practical feasibility of using GPUs for intra-operative real-time navigation and monitoring.

## Supporting Information

S1 Text **This file contains source code and data repository locations.**
(TXT)
